# Kyste synovial intraosseux du scaphoïde carpien bilatéral révélé par une fracture pathologique: à propos d'un cas et revu de la littérature

**DOI:** 10.11604/pamj.2015.21.315.6451

**Published:** 2015-08-28

**Authors:** Merouane Abouchane, Amine Belmoubarik, Hamza Benameur, Mohammed Nechad

**Affiliations:** 1 Service de Chirurgie Traumatologique et Orthopédique Aile 4, CHU Ibn Rochd, Casablanca, Maroc

**Keywords:** Scaphoïde, kyste, bilatéral, fracture pathologique, scaphoid, cyst, bilateral, pathological fracture

## Abstract

Nous rapportons l'observation d'un jeune patient qui présente un kyste synovial intraosseux (KSIO) du scaphoïde révélé par une fracture pathologique. Le kyste synovial intraosseux du scaphoïde constitue une étiologie très rare des douleurs du poignet encore plus des fractures et la forme bilatérale associe à une fracture demeure une entité exceptionnelle, non décrite dans la littérature.

## Introduction

Le KSIO est une rare source de douleur au niveau du carpe, et a été décrit au niveau du scaphoïde [1], semi lunaire [2], le capitatum [3], le triquetrum [4], l'hamatum et le pisiforme [5, 6]. La forme bilatérale au niveau du carpe été exceptionnellement décrite, encore plus au niveau scaphoïde [1, 7] par contre aucun cas n'a été rapporté associant une fracture pathologique.

## Patient et observation

Nous rapportons le cas de Mr. M.E jeune patient de 29 ans, serveur dans un café, sans antécédent particulier, qui a présenté suite à une chute de sa moto avec réception sur la paume de la main gauche (non dominante) traumatisme fermé du poignet et de la main gauche. A l'examen clinique, retrouve douleur du poignet, œdème de la tabatière anatomique et douleur provoquée à la traction du premier rayon, associé à une douleur à la palpation de la face dorsale de la main gauche. Toutefois, il nous signalait qu'il avait ressenti avant son traumatisme et en l'absence d'antécédent traumatique des douleurs occasionnelles d'effort du poignet gauche comme au poignet droit, ce qui nous a alertés a demandé la radio du poignet droit.

### La radiographie standard

**Figure 1:**
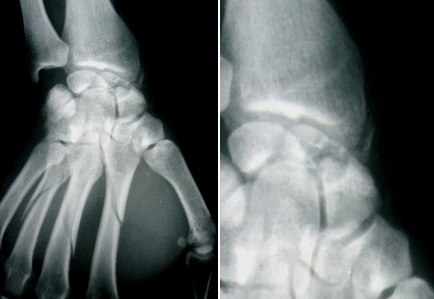
Fracture pathologique du scaphoïde gauche sur KSIO avec fracture du 2e, 3e et 4e métacarpien

**Figure 2:**
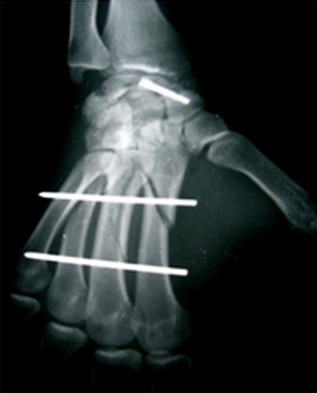
KSIO du scaphoïde droit

**Figure 3:**
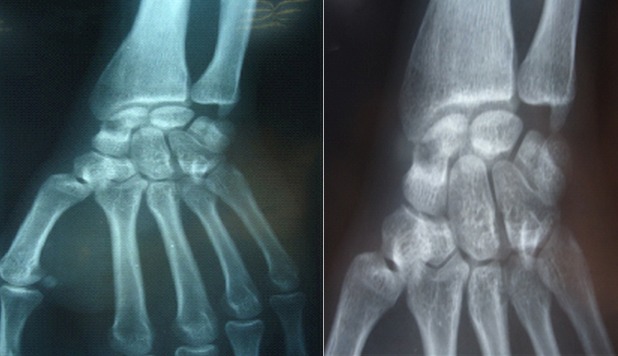
Radio de contrôle: vissage scaphoïdien et brochage transversal des métacarpiens

**Figure 4:**
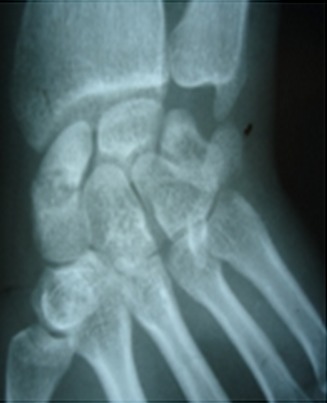
Radio post opératoire après curetage et comblement

**Figure 5:**
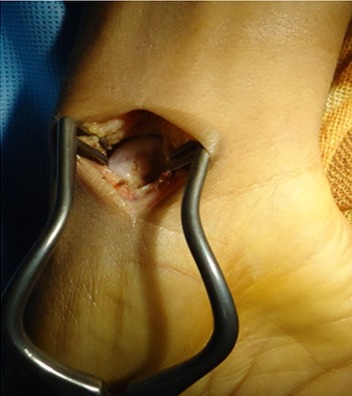
Contenu mucoïde jaunâtre

Du poignet gauche (face et profil) révèle une image lacunaire polycyclique bien limité sans caractère agressif, localisée au niveau de la partie moyenne du scaphoïde (col), bien délimitée ne soufflant pas l'os, interrompue par une rupture corticale (fracture). La fracture n’était pas déplacée, associée à des fractures spiroides des 2^°^, 3^°^ et 4^ème^ métacarpiens ipsilaterale (Figure 1). Du poignet droit révèle une géode excentrée en externe en regard du tubercule du scaphoïde soufflant légèrement la corticale externe sans signe d'arthrose péri-scaphoïdienne ni de communication apparente avec les articulations contigus (Figure 2). Le patient a été opéré 4 jours après sa consultation pour les deux poignets après avoir pris son consentement pour aborder le poignet droit. A gauche: Une seule voie d'abord dorsale était nécessaire, avait révélé une fracture corporéale du scaphoïde avec une tumeur de contenu mucoïde jaunâtre. Nous avions réalisé un évidement avec comblement par de l'os spongieux prélevé de l'extrémité distale du radius. L'ostéosynthèse de la fracture du scaphoïde était réalisée par vissage de Herbert, les fractures des métacarpiens étaient ostéosynthèsèes par double embrochage transversale à l'aide de broches de Kirchner 14/10 (Figure 3). Une manchette plâtrée prenant la colonne du pouce en opposition jusqu’à la consolidation qui a été obtenue 45 jours après l'intervention, aux termes desquels les deux broches ont été retirées. Les soins postopératoires ont ensuite comporté une rééducation fonctionnelle de 1 mois et demie. Les suites opératoires ont été simples. A droite: une voie palmaire a été pratique retrouvant une géode intrascaphoïdienne bordée par une mince membrane fibreuse et contenant un liquide mucoïde jaunâtre (Figure 4) la cavité est curetée et comblée de greffons spongieux prélevé au niveau de la métaphyse radiale distale ipsilaterale (Figure 5). L'examen histologique conforme le diagnostic de kyste synovial intraosseux. L'examen bactériologique été négatif. Au dernier recul à une année et demie après le traitement, le patient a récupéré une bonne fonction du poignet avec indolence et reprise de ses activités de loisir et professionnelle.

## Discussion

Le KSIO du carpe est beaucoup moins fréquent que celui des os longs, qui se localise essentiellement en sous chondral et en juxtaprticulaire [1]. Quatre des 88 patients présentant un KSIO rapportes par Schajowics et al [1] avaient des localisations bilatérales, une fois au niveau du semi lunaire et une fois au niveau du scaphoïde, Samuel et al rapportent un cas de localisation bilatérale au niveau du scaphoïde [1], Kligman lui décrit un cas original avec localisation simultanée bilatérale au niveau du semi lunaire et le scaphoïde [8]. Mais a aucun cas n'a été rapporte décrivant une localisation bilatérale associée a une fracture pathologique. La plupart des KSIO se développent au moyen âge, avec une moyenne d’âge dans les deux plus grandes séries de 41 et 47 ans [1]. La physiopathogénie reste controversée. Schématiquement deux hypothèses principales s'affrontent. Certains auteurs pensent que le kyste osseux se forme par inclusion synoviale de dehors en dedans. D'autres envisagent une métaplasie synoviale débutant au sein même de l'os, éventuellement favorisée par des phénomènes microtraumatiques ou ischémiques locaux [8, 9] La lésion peut être: asymptomatique et mise en évidence à l′occasion de radiographies standards [10]; douleurs habituellement modérées mais répondant souvent mal aux antalgiques. Ces douleurs pourraient être expliquées par l'hyperpression intra-osseuse secondaire au développement d'un processus pathologique dans une cavité restreinte inextensible [11]; tuméfaction du poignet suite a une rupture du KSIO et la diffusion de son continu en intra-articulaire [12]; fracture pathologique responsable d'une recrudescence des douleurs [1]. Jusqu’à aujourd'hui aucun cas de KSIO de scaphoïde bilatéral n'a été révélé par une fracture pathologique. La cause de cette localisation bilatérale reste incertaine [1], qui peut être en relation avec une variante anatomique bilatérale possiblement en rapport avec une insertion défectueuse du ligament radioscapholunaire favorisant une collection du liquide articulaire lors de sa course anatomique [1] Devant une symptomatologie invalidante et réfractaire, l'indication chirurgicale semble s′imposer [10]. Ce n'est que devant une symptomatologie invalidante et réfractaire pendant une durée d'au moins de six mois [13] ou l'apparition de signes radiologiques d’évolutivité que l'indication chirurgicale sera posée [11, 13, 14]. L'intervention consiste en une exérèse chirurgicale, avec une greffe osseuse spongieuse autologue et un moyen de synthèse si cela est nécessaire [15]. La greffe osseuse vascularisée aux dépens de l'artère transverse antérieure du carpe se déroulant sur le même site, constitue une autre alternative chirurgicale [12]. Le pronostic fonctionnel est généralement bon, et la récidive est exceptionnelle [12].

## Conclusion

Le kyste synovial intraosseux du scaphoïde bilatérale est une lésion géodique bénigne rare. Elle peut être découverte, par hasard ou rarement, par des douleurs du poignet, exceptionnellement par une fracture comme c'est le cas de notre observation. Nous attirons l'attention sur l'intérêt de l'interrogatoire, l'examen clinique et la radiologie comparative.
